# Strength in numbers: achieving greater accuracy in MHC-I binding prediction by combining the results from multiple prediction tools

**DOI:** 10.1186/1745-7580-3-5

**Published:** 2007-03-24

**Authors:** Brett Trost, Mik Bickis, Anthony Kusalik

**Affiliations:** 1 Departments of Computer Science and Mathematics & Statistics, University of Saskatchewan, Saskatchewan, Canada

## Abstract

**Background:**

Peptides derived from endogenous antigens can bind to MHC class I molecules. Those which bind with high affinity can invoke a CD8^+ ^immune response, resulting in the destruction of infected cells. Much work in immunoinformatics has involved the algorithmic prediction of peptide binding affinity to various MHC-I alleles. A number of tools for MHC-I binding prediction have been developed, many of which are available on the web.

**Results:**

We hypothesize that peptides predicted by a number of tools are more likely to bind than those predicted by just one tool, and that the likelihood of a particular peptide being a binder is related to the number of tools that predict it, as well as the accuracy of those tools. To this end, we have built and tested a heuristic-based method of making MHC-binding predictions by combining the results from multiple tools. The predictive performance of each individual tool is first ascertained. These performance data are used to derive weights such that the predictions of tools with better accuracy are given greater credence. The combined tool was evaluated using ten-fold cross-validation and was found to signicantly outperform the individual tools when a high specificity threshold is used. It performs comparably well to the best-performing individual tools at lower specificity thresholds. Finally, it also outperforms the combination of the tools resulting from linear discriminant analysis.

**Conclusion:**

A heuristic-based method of combining the results of the individual tools better facilitates the scanning of large proteomes for potential epitopes, yielding more actual high-affinity binders while reporting very few false positives.

## Background

The major histocompatibility complex (MHC) is a set of genes whose products play a crucial role in immune response. Peptides derived from the proteasomal degradation of intracellular proteins are presented by MHC class I molecules to cytotoxic T lymphocytes (CTL) [1-3], and recognition of a non-self peptide by a CTL can result in the destruction of an infected cell. Peptides that can complete this pathway are called T cell epitopes.

Only 0.5% of peptides are estimated to bind to a given MHC-I molecule, making this the most selective step in the recognition of intracellular antigens [4,5]. Given the large size of many viral and bacterial proteomes, it is prohibitive in terms of time and money to test every possible peptide for immunogenicity; thus, tools for the computational prediction of peptides that are likely to bind to a given MHC-I allele are invaluable in facilitating the identification of T cell epitopes.

Many tools for performing such predictions, of varying quality, are available. We hypothesize that greater predictive accuracy can be achieved by combining the predictions from several of these tools rather than using just one tool. Further, contributions from individual tools should be related to their accuracy. To test this hypothesis, we have built a prediction tool which assigns a "combined score" to each peptide in a given protein by taking into account the predictive performance of each tool, and the score given by that same tool to a given peptide. We also compare our technique with combined predictions made using linear discriminant analysis, a standard statistical method for combining variables to distinguish two groups (in this case, "binder" and "non-binder"). In this paper, the acronym "HBM" will refer to our heuristic-based method and "LDA" will refer to the predictor built using linear discriminant analysis.

## Results and discussion

### Performance of the individual tools

Table 1 shows the ability of each individual tool to discrimine between the binders and nonbinders to HLA-A*0201 derived from the community binding database [6]. As we are interested in good sensitivity at high specificity, the sensitivity of each tool at 0.99 specificity and 0.95 specificity are shown. The *A*_*ROC *_value for each tool is also given; these values are very similar, but not completely identical, to those given by the authors of the community binding resource; the small discrepancies are likely due to the use of differing methods of calculating the area under the ROC curve. Individual tool performance data for the HLA-B*3501 and H-2Kd peptides from the community binding database, as well as for the HLA-A*0201 peptides gathered from the literature, are shown in Tables 2, 3, and 4, respectively.

**Table 1:** Performances of the individual prediction tools on the HLA-A*0201 peptides from the community binding resource.

Tool	*A*_*ROC*_	*0.99 Specificity*			*0.95 Specificity*		
		
		Rank^1^	Sensitivity	Threshold^2^	Rank	Sensitivity	Threshold
ARB Matrix	0.935	4	0.188	2.190	2	0.601	42.950
NetMHC 2.0 ANN	0.932	1	0.286	153.000	1	0.611	920.000
SMM	0.922	2	0.201	38.092	4	0.543	454.865
Bimas	0.920	3	0.198	324.068	3	0.552	47.991
SYFPEITHI	0.885	8	0.170	27.000	5	0.421	24.000
Multipred ANN	0.884	9	0.140	5.820	7	0.373	5.560
NetMHC 2.0 Matrix	0.872	6	0.177	24.329	10	0.358	20.129
SVMHC MHCPEP	0.870	12	0.115	1.000	11	0.334	0.520
Logistic Regression	0.862	13	0.101	0.364	9	0.364	0.108
SVMHC SYFPEITHI	0.854	7	0.176	0.950	8	0.367	0.490
HLA Ligand	0.825	10	0.137	141.000	14	0.274	127.000
Rankpep	0.822	15	0.077	103.000	13	0.306	83.000
MHCPred (Interactions)	0.818	5	0.182	43.350	6	0.377	99.080
MHCPred (position only)	0.814	11	0.116	21.330	12	0.311	65.310
Multipred HMM	0.798	14	0.090	7.530	15	0.244	7.090
Predep	0.788	16	0.045	-6.000	16	0.217	-5.130

The predictive performance of each tool for the HLA-A*0201 community binding data is shown using two measures: *A*_*ROC *_score, and sensitivity when specificity is 0.99 and 0.95. ^1^Indicates how the sensitivity of each tool compares to that of the other tools at the indicated specificity; the tool with rank 1 has the highest sensitivity. ^2^The scoring threshold corresponding to the indicated specificity.

**Table 2:** Individual tool *A*_*ROC *_values and sensitivity data for HLA-B*3501 using binders and nonbinders from the community binding resource.

Tool	*A*_*ROC*_	*0.99 Specificity*			*0.95 Specificity*		
		
		Rank	Sensitivity	Threshold	Rank	Sensitivity	Threshold
ARB Matrix	0.849	1	0.242	12.890	1	0.422	296.090
Bimas	0.808	7	0.047	60.000	8	0.166	24.000
NetMHC 2.0 Matrix	0.789	2	0.137	32.386	2	0.336	28.404
HLA Ligand	0.786	8	0.043	162.000	4	0.237	145.000
Rankpep	0.769	5	0.071	124.000	3	0.256	107.000
Logistic Regression	0.764	11	0.024	0.655	11	0.123	0.259
SVMHC SYFPEITHI	0.742	3	0.118	0.680	6	0.204	0.420
SVMHC MHCPEP	0.733	8	0.043	1.560	6	0.204	1.140
MHCPred (position only)	0.692	6	0.057	140.600	9	0.156	210.860
MHCPred (interactions)	0.683	4	0.090	52.240	5	0.209	179.470
Predep	0.587	10	0.038	-6.020	10	0.128	-5.090

For details, see the caption for Table 1.

**Table 3:** Individual tool *A*_*ROC *_values and sensitivity data for H-2Kd using binders and nonbinders from the community binding resource.

Tool	*A*_*ROC*_	*0.99 Specificity*			*0.95 Specificity*		
		
		Rank	Sensitivity	Threshold	Rank	Sensitivity	Threshold
SYFPEITHI	0.918	3	0.300	27.000	4	0.483	25.000
ARB Matrix	0.899	1	0.500	15.470	1	0.583	40.800
Bimas	0.888	4	0.183	4800.000	3	0.533	2880.000
Rankpep	0.886	2	0.317	107.000	2	0.567	98.000

For details, see the caption for Table 1.

**Table 4:** Individual tool *A*_*ROC *_values and sensitivity data for HLA-A*0201 using binders and nonbinders gathered from the literature

Tool	*A*_*ROC*_	*0.99 Specificity*			*0.95 Specificity*		
		
		Rank	Sensitivity	Threshold	Rank	Sensitivity	Threshold
Multipred ANN	0.772	11	0.083	5.830	2	0.278	5.600
NetMHC 2.0 ANN	0.772	6	0.139	99.000	7	0.231	300.000
SYFPEITHI	0.762	1	0.194	27.000	2	0.278	25.000
SVMHC MHCPEP	0.745	1	0.194	0.910	4	0.269	0.740
Logistic Regression	0.743	10	0.093	0.424	12	0.167	0.281
ARB Matrix	0.742	8	0.102	1.860	14	0.139	3.550
Bimas	0.722	12	0.074	437.482	9	0.213	159.970
NetMHC 2.0 Matrix	0.719	14	0.046	27.193	10	0.194	23.585
SMM	0.716	4	0.157	100.484	6	0.250	262.434
Rankpep	0.708	3	0.176	89.000	5	0.259	83.000
MHCPred (interactions)	0.707	6	0.139	61.660	1	0.287	100.690
SVMHC SYFPEITHI	0.706	5	0.148	0.970	11	0.185	0.820
HLA Ligand	0.705	12	0.074	147.000	12	0.167	138.000
MHCPred (position only)	0.700	8	0.102	28.250	7	0.231	67.300
Multipred HMM	0.695	16	0.009	7.570	15	0.102	7.290
Predep	0.627	15	0.019	-6.450	16	0.093	-5.330

For details, see the caption for Table 1. The peptides in this literature-derived dataset are available in Additional File 1.

### Performance of the combined methods

The HBM and LDA were evaluated using ten-fold cross-validation on the same four datasets (the HLA-A*0201, HLA-B*3501, and H-2Kd datasets from the community binding resource, and the HLA-A*0201 dataset from the literature) as the individual tools.

The HBM requires that an individual tool specificity parameter be chosen such that the tools' sensitivities at that specificity can be used as the weights in equation 1. The performance of the HBM was determined using individual tool specificities of 0.99, 0.95, 0.90, and 0.80. In general, it was found that using 0.99 individual tool specificity resulted in the best performance, while the use of lower individual tool specificity parameters resulted in somewhat weaker performance. Thus, all of the HBM performance data described below were obtained using 0.99 individual tool specificity. Table 5 shows the performance of the HBM on all four datasets.

**Table 5:** Performance of the heuristic-based method on all four datasets

Specificity	HLA-A*0201 (comm)	HLA-B*3501 (comm)	H-2Kd (comm)	HLA-A*0201 (lit)
0.99	0.404	0.313	0.467	0.271
0.95	0.618	0.393	0.617	0.475

The sensitivity of the HBM is shown at 0.99 specificity and 0.95 specificity for all four of the datasets used in this study. All values were obtained using a value of 0.99 for the individual tool specificity parameter. The abbreviation "comm" refers to peptides derived from the community binding database, while "lit" refers to peptides gathered from the literature.

For two of the three alleles, the HBM showed marked improvements in sensitivity at high specificity compared with the best-performing individual tools. The sensitivity of the HBM at 0.99 specificity for HLA-A*0201 was 0.40, a large increase over NetMHC ANN, whose sensitivity of 0.29 was the best among the individual tools. For HLA-B*3501, the HBM sensitivity was 0.31 at a specificity of 0.99, while the highest sensitivity obtained by an individual tool was 0.24. The HBM showed similarly strong performance when tested using the literature-derived HLA-A*0201 data, achieving a sensitivity of 0.27, compared with 0.19 for the best-performing individual tool. For H-2Kd, however, the HBM was outperformed at 0.99 specificity by the ARB matrix tool, which had a sensitivity of 0.50 versus 0.47 for the HBM. We note, however, that ARB Matrix was trained using binders from the community binding database, so its performance on the community datasets is likely inflated [7]

At lower specificity thresholds, the advantage of the HBM was only marginal. For instance, the sensitivity of the HBM at 0.95 specificity for the HLA-A*0201 community dataset was almost identical to that of the best individual tool; for HLA-B*3501, the sensitivity of the HBM at specificity 0.95 was slightly worse than the individual tool with the highest sensitivity at that specificity. Interestingly, however, the HBM actually outperforms the individual tools at specificity 0.95 for H-2Kd.

**Figure 1:**
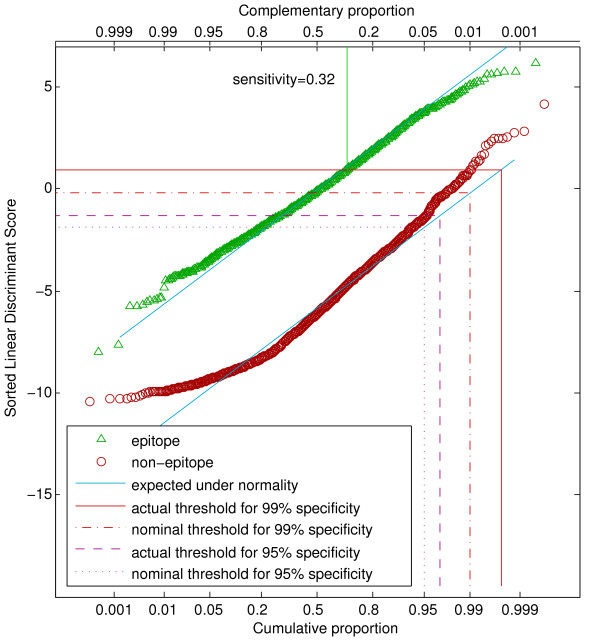
Q-Q plot showing distribution of LDA scores for the HLA-A*0201 community data set. The horizontal axis has been scaled according to normal probabilities, so that points from a normally distributed variable would fall along a straight line (shown in blue). Scores lying above a threshold indicated by a horizontal line would be classified as epitopes. A level exceeding 99% of a normal distribution defines a nominal specificity of 0.99, whereas an actual specificity of 0.99 requires a threshold meeting the actual distribution of points at the 0.99 vertical line. The realized sensitivity of 0.32 for a specificity of 0.99 is indicated as the proportion of epitopes whose scores lie above the threshold of 0.95.

The linear discriminant scores displayed approximately normal distributions, with moderate separation between binders and non-binders. The distributions were closer to normality for HLA-A*0201 dataset from the literature and the H-2Kd datset, with more systematic deviations for the other two datasets. While the nominal sensitivity and specificity of the LDA agreed reasonably well with the actual and cross-validated values, we used the cross-validated values for comparison purposes (Table 6). The distinction between nominal and actual specificity is illustrated in Figure 1.

**Table 6:** Performance of linear discriminant analysis on all four datasets

Specificity	HLA-A*0201 (comm)	HLA-B*3501 (comm)	H-2Kd (comm)	HLA-A*0201 (lit)
0.99	0.324	0.213	0.417	0.102
0.95	0.718	0.436	0.633	0.333

*A*_*ROC*_	0.956	0.885	0.935	0.828

The sensitivity of the combined tool is shown at 0.99 specificity and 0.95 specificity for all four of the datasets used in this study. The abbreviation "comm" refers to peptides derived from the community binding database, while "lit" refers to peptides gathered from the literature.

LDA displayed an improvement over the individual tools for the HLA-A*0201 community dataset, attaining a sensitivity of 0.33 at 0.99 specificity – higher than that of all the individual tools, but lower than that of the HBM. The performance of the LDA on the other datasets was less substantial. Its sensitivity on the HLA-A*3501 communtiy data at 0.99 specificity was 0.21, compared to 0.24 for ARB matrix and 0.31 for the HBM. However, we note again that the ARB matrix sensitivity is probably inflated, especially considering that the sensitivity for the second-best tool at 0.99 specificity (NetMHC 2.0 Matrix) was 0.14. The performance of LDA on the H-2Kd dataset was fairly strong, but still lower than that of both ARB Matrix and the HBM. Finally, the performance of LDA on the literature-derived HLA-A*0201 dataset was fairly weak at both 0.99 specificity and 0.95 specificity.

Purely in terms of the *A*_*ROC *_value, however, LDA outperforms the individual tools on all four datasets. This suggests that while LDA provides strong "overall" performance across the entire spectrum of specificities, it achieves less improvement in the region of the ROC curve that is of interest in this study – namely, the regions of very high specificity.

## Discussion

In this paper, results are given only for the three alleles HLA-A*0201, HLA-B*3501, and H-2Kd. The approach can be easily extended to any arbitrary MHC-I allele, provided that a sufficient number of tools make predictions for that allele, and that there exists an adequate number of known binding and non-binding peptides that can be used to test the individual tools on that allele. The effects of the latter conditions are born out in our results for H-2Kd versus HLA-A*0201.

We have used our HBM tool for the prediction of binders from bench-lab experiments, with positive results. For instance, in predicting binders for influenza virus in mice, the best two 9-mers predicted by HBM turned out to generate the strongest responses in immunoassays [8].

Some comparative studies of binding prediction tools use randomly-generated nonbinders. This study used known nonbinders only. We contend that the use of known nonbinders contributes to a stronger practical assessment of each tool's utility. Such nonbinders that might have been selected by an experimenter for binding-affinity testing due to the presence of good anchor residues. Randomly-generated nonbinders tend to have anchor residues that poorly match established motifs, and thus are typically very easy to classify; in contrast, nonbinders reported in the literature frequently have anchor residues that do conform to an established motif, making them more difficult to classify. For a tool to be truly useful, it must be able to differentiate between peptides that all have good anchor residues, but whose non-anchor residues confer different degrees of binding affinity.

### Availability

The authors have elected not to make the HBM available online, for two reasons: first, frequent server outages and other problems with individual web-based tools often prevent acquisition of all the requisite scores. Automatic operation is therefore not possible. Second, the querying of all the web-based tools can take a long time, making the tool inconvenient for real-time web-based access. Interested researchers may, however, contact the authors regarding obtaining the scripts implementing the HBM.

## Conclusion

We have built a tool that heuristically combines the output of several individual MHC-binding prediction tools, and have shown that it achieves substantially improved sensitivity at high specificity compared to the best individual tools, and is also superior to linear discriminant analysis at high specificity. This technique is very general, and can be updated as new prediction tools become available. Given this, the HBM should be extremely valuable for researchers wishing to scan large proteomes for potential epitopes. Additionally, the combination of the tools using linear discriminant analysis consistently displays improved overall operating characteristics (as measured by the *A*_*ROC *_value) over the individual tools, and thus would be useful for researchers desiring to identify a large number of the potential binders in a smaller dataset, such as a single protein.

The success of our heuristic-based tool substantiates the hypothesis that peptides predicted by a number of tools is more likely to bind than those predicted by just one tool, and that the likelihood of a particular peptide being a binder is related to the number of tools that predict it, as well as the accuracy of those tools. In the same vein, our data suggests that the performance of the heuristic-based approach improves when more individual prediction tools are available. The fact that combining the output of several tools results in increased performance indicates that, as of now, no single tool is able to extract all the information inherent in the data currently available. Thus, continued work on improved MHC-binding prediction is necessary.

## Methods

### Determination of prediction tools

We have identified a total of 16 different prediction tools from 12 different research groups. Where there are two tools from the same group, they differ either in the method used to predict binding affinity or in the data used to train the model. The tools tested are as follows: Bimas [9], Rankpep [10], SYFPEITHI [11], NetMHC 2.0 ANN and NetMHC 2.0 Matrix [5,12,13], SVMHC SYFPEITHI and SVMHC MHCPEP [14], HLA Ligand [15], Predep [16], SMM [2], MHCPred (position only) and MHCPred (interactions) [17,18], Multipred HMM and Multipred ANN [19-21], ARB Matrix [7], and a locally implemented logistic regression-based tool [22].

### Creating a collection of peptides for evaluating the predictive performance of each tool

Prediction of peptide binding was evaluated for three different alleles: HLA-A*0201, HLA-B*3501, and H-2Kd. These alleles differ substantially in the number of available tools that make predictions for them: all of the aforementioned tools predict for HLA-A*0201, eleven make predictions for HLA-B*3501, and just four predict for H-2Kd. Thus, these alleles were chosen so that the performance of our combined tool (HBM) and linear discriminate analysis (LDA) could be evaluated when different numbers of individual tools are employed.

Two sources of data were used for comparative analysis of prediction tools in this study. The first was the community binding resource [6], a large, recently published database containing experimentally determined affinity values for the binding of peptides to many different MHC-I alleles. This dataset of testing peptides could potentially be expanded further by incorporating peptides from such online databases as SYFPEITHI [11], MHCPEP [23], HLA Ligand [15], and EPIMHC [24]. However, the use of the latter online databases presents a problem for the current study. As the models underlying many existing prediction tools were trained using data from these latter databases, the subsequent testing of the individual tools with these same peptides may result in an inaccurate estimation of each tool's predictive performance. For instance, tool A may be judged better than tool B merely because tool A was trained using the same peptides with which it was tested, while tool B was not. As combining the scores of the individual tools relies on an accurate appraisal of the performance of each tool, it is necessary to avoid the use of peptides with which the individual tools have been trained. Thus, we used only the community binding resource as our source of binding-affinity data. Only peptides of length 9 were considered, because all tools make predictions for peptides of this length. Peptides with *IC*_50 _< 500 nM were classified as binders, while those having *IC*_50 _> 500 nM were classified as nonbinders. In total, there were 1184 binders and 1905 non-binders to HLA-A*0201, 211 binders and 525 nonbinders to HLA-A*3501, and 60 binders and 116 nonbinders to H-2Kd.

For comparison purposes, the tools were also tested using an independent dataset consisting of peptides gathered only from published literature [25-33]. Again, only nonamers were chosen. Classifying a given peptide as a binder or a nonbinder was performed as follows: if *IC*_50 _values were reported (as in the community binding database and most literature sources), then the standard binding threshold of 500 nM was used; where some other type of assay was done to determine binding affinity, the classification given by the authors was used. In the latter case, if no classification was given by the authors, the peptides were not used. Finally, to avoid bias in the data, peptides were filtered such that where two peptides differed at fewer than two residues, one peptide was randomly removed. The resultant dataset consisted of 108 binders and 108 nonbinders to HLA-A*0201, and are given in Additional File 1. Due to scarcity of published data, it was not possible to construct similar datasets for HLA-B*3501 or H-2Kd.

### Performance measures

Binding prediction programs give a numeric score to each considered peptide. Each score can be converted to a binary prediction by comparing against a tool-specific threshold – if the score is greater or equal, then the peptide is a predicted binder; otherwise, it is a predicted nonbinder.

Sensitivity is the proportion of experimentally determined binders that are predicted as binders and is defined as *true positives*/(*true positives + false negatives*). Specificity is the proportion of experimentally determined nonbinders that are predicted as nonbinders, and is defined as *true negatives*/(*true negatives + false positives*). The traditional way to measure the performance of a classifier is to use a receiver operating characteristic (ROC) curve. However, ROC curves do not always give a good measure of practical utility. For a researcher scanning a large proteome for potential epitopes, specificity may be much more important than sensitivity. Imagine scanning a proteome consisting of 10,000 overlapping nonamers, 50 of which (unbeknownst to the experimenter) are good binders to the MHC-I allele of interest. Consider further that prediction tool A has 0.70 sensitivity at 0.80 specificity and 0.05 sensitivity at 0.99 specificity.

Tool B has 0.50 sensitivity at 0.80 specificity and 0.20 sensitivity at 0.99 specificity. While tools A and B might have the same area under the ROC curve (*A*_*ROC*_), tool A is superior at 0.80 specificity and tool B is superior at 0.99 specificity. If tool A is used at a threshold corresponding to 0.80 specificity, then approximately 2000 peptides must be tested in order to find 35 of the high-affinity binders. In contrast, if tool B is used at a threshold corresponding to 0.99 specificity, only about 100 peptides would have to be tested in order to find 10 of the high-affinity binders. Due to the high cost of experimental testing, and because knowledge of all the binders in a given proteome is usually not needed, the latter scenario would be preferable. We therefore conclude that good sensitivity at very high specificity is a more practical measure of a tool's usefulness than the *A*_*ROC *_value, and have thus used sensitivity at high values of specificity as the primary assessor of the practical utility of each tool. For completeness, however, we also include each tool's *A*_*ROC *_value.

### Combining the scores of the individual tools

We propose a heuristic-based method (HBM) for combining scores from individual prediction tools to make a better prediction. This method takes advantage of the observation that most of the individual tools make very few false positive predictions when the classification threshold is set sufficiently high, but correspondingly make few predictions of positives. If the tools identify different actual binders, combining such predictions may result in a greater number of rrue positives. The method also tries to take advantage of the "collective wisdom" of a group of predictive tools. The individual tools are based on a variety of techniques. Instead of trying to find the "best" technique, we try to combine the best that each technique has to offer. This is an extension of the idea used by prediction tools such as MULTIPRED [19] which combine predictions made by a few methods.

Our proposed combined prediction tool ("HBM") takes a protein sequence as input, queries all of the individual prediction tools getting from each the predicted binding affinity for all nonamers in the protein, computes a combined score for each nonamer, and finally predicts binders based on the combined scores for all nonamers. The tool is implemented as a Perl script.

The first step in our HBM is to select a specificity for the individual tools. Each tool is then weighted according to its sensitivity at that specificity. Next, the score given to each peptide by a given prediction tool is compared to the tool-specific threshold value for that specificity. If the score is better than or equal to the threshold score, then that tool predicts the peptide as a binder, and the weight (sensitivity at the chosen specificity) for that tool is added to the total score for the peptide. Otherwise, the peptide's total score remains unchanged. For peptide *x *and each prediction tool *t*, we have

CombinedScore(x)=∑tBt(x)Wt     (1)
 MathType@MTEF@5@5@+=feaafiart1ev1aaatCvAUfKttLearuWrP9MDH5MBPbIqV92AaeXatLxBI9gBaebbnrfifHhDYfgasaacH8akY=wiFfYdH8Gipec8Eeeu0xXdbba9frFj0=OqFfea0dXdd9vqai=hGuQ8kuc9pgc9s8qqaq=dirpe0xb9q8qiLsFr0=vr0=vr0dc8meaabaqaciaacaGaaeqabaqabeGadaaakeaacqqGdbWqcqqGVbWBcqqGTbqBcqqGIbGycqqGPbqAcqqGUbGBcqqGLbqzcqqGKbazcqqGtbWucqqGJbWycqqGVbWBcqqGYbGCcqqGLbqzcqGGOaakcqWG4baEcqGGPaqkcqGH9aqpdaaeqbqaaiabdkeacnaaBaaaleaacqWG0baDaeqaaOGaeiikaGIaemiEaGNaeiykaKIaem4vaC1aaSbaaSqaaiabdsha0bqabaaabaGaemiDaqhabeqdcqGHris5aOGaaCzcaiaaxMaadaqadaqaaiabigdaXaGaayjkaiaawMcaaaaa@51F5@

where *B*_*t*_(*x*) is 1 if peptide *x *is predicted to bind by tool *t *and 0 otherwise, and *W*_*t *_is the weight of tool *t*. CombinedScore(*x*) is then compared to a threshold in order to classify *x *as either a predicted binder or a predicted nonbinder.

The performance of the HBM was determined using 10-fold cross-validation: in each fold, 90% of the peptides (the "training peptides") were used to determine the performances of the individual tools, and these performance data were used by the HBM as described above to make predictions for the remaining 10% (the "testing peptides"). Each peptide was used as a testing peptide exactly once. The scores given to each testing peptide were then used to calculate specificity and sensitivity values for the HBM in the same manner as was described for the individual tools. To minimize experimental error due to the random partitioning of the peptides into training and testing sets, the entire process described above was repeated ten times, and the HBM sensitivity at each specificity was taken to be the average of its sensitivity in the ten trials. While *A*_*ROC *_values are shown for the individual tools and for the LDA, no such values could be computed for the HBM. The reason for this is that, at high individual tool specificity parameters, most nonbinding peptides get an HBM score of zero, and therefore the ROC curve contains no points for specificities between 0 and approximately 0.85–0.90.

### Comparison technique

A standard method for combining variables to distinguish two categories is linear discriminant analysis (LDA) [34]. If *y *is the vector of scores from all the tools for a particular peptide, it is classified according to the value of the linear discriminant

(*μ*_1 _- *μ*_0_)'∑^-1^*y*,

where *μ*_0 _and *μ*_1 _are the vectors of means for non-epitopes and epitopes, respectively, and ∑ is the average covariance matrix of the scores within the two groups. This method is optimal (in the sense of minimizing the probability of misclassification) if the scores have a multivariate normal distribution with the same covariance matrix for epitopes and non-epitopes. More sophisticated methods have been developed without the normality assumption, but doubts have been expressed about their advantage [35]. The separation between the groups can then be quantified by

*δ*^2 ^= (*μ*_1 _- *μ*_0_)'∑^-1^(*μ*_*1 *_- *μ*_0_).

Under the normality assumption, if the specificity is fixed at 1 - *α*, then the sensitivity will be

Φ (*δ *+ Φ^-1^(*α*)),

where Φ is the cumulative distribution function (cdf) of the standard normal distribution. *A*_*ROC *_can be calculated as Φ (*δ*/2
 MathType@MTEF@5@5@+=feaafiart1ev1aaatCvAUfKttLearuWrP9MDH5MBPbIqV92AaeXatLxBI9gBaebbnrfifHhDYfgasaacH8akY=wiFfYdH8Gipec8Eeeu0xXdbba9frFj0=OqFfea0dXdd9vqai=hGuQ8kuc9pgc9s8qqaq=dirpe0xb9q8qiLsFr0=vr0=vr0dc8meaabaqaciaacaGaaeqabaqabeGadaaakeaadaGcaaqaaiabikdaYaWcbeaaaaa@2DB9@). The threshold for classification is determined by the prior probability *p*_1 _that a peptide is an epitope, which is related to the specificity by

*p*_1 _= [1 + exp (-*δ*^2^/2 - *δ*Φ^-1^(*α*))]^-1^.

A number of the tools displayed notably non-normal distributions. Most of these were highly skewed, but became close to normal when transformed to logarithms. The scores of three tools (NetMHC 2.0 ANN, Multipred ANN, and the logistic regression-based tool) had sigmoidal distributions. These became approximately normal when converted to scaled logits. A "logit" is a transformation of a probability *p *(between 0 and 1) to log(*p*/(1 - *p*)). For a variable *y *which is restricted between *a *and *b*, a "scaled logit" can be calculated via log((*y *- *a *+ *ε*)/(*b *- *y *- *δ*)), where *ε *and *δ *are small adjustments to avoid zeros. *ε *= (*y*_- _- *a*)/2 and *δ *= (*b *- *y*_+_)/2, *y*_- _and *y*_+ _being the smallest and largest observed values greater or less than *a *or *b*, respectively. The actual performance of the linear discriminant on the transformed scores was estimated using ten-fold cross-validation. Computations were done using S-PLUS version 7.0.0. Figures were created with MATLAB 7.

Except for the H-2Kd dataset, the cross-validated specificities fell short of the nominal ones. To realize specificities of 0.99 and 0.90, the threshold was adjusted to a nominal specificity such that the cross-validated values were as close as possible to the target values. Figure 1 shows the distributions of the LDA scores for the community HLA-A*0201 data set. The diagonal lines indicate where the points are expected to fall for perfectly normal data. A specificity of 0.99 corresponds to a horizontal line such that 99% of the non-epitopes fall below this line. Because of the slight upward curvature of the non-epitope distribution, a nominal specificity of 0.99 falls short of this goal, but the larger nominal value of 0.9975 gives the correct threshold. About 32% of the epitopes give LDA scores above this value. Distributions of LDA scores for the the other datasets are given in Additional Files 2, 3 and 4.

## Abbreviations

LDA – linear discriminant analysis

HBM – heuristic-based method

## Competing interests

The authors have no conflict-of-interest with respect to this work. In particular, they have no direct connection with any of the researchers involved with the binding prediction tools studied.

## Authors' contributions

BT performed the design, programming work, and evaluation of the HBM. MB performed the linear discriminate analysis work. AK proposed the original idea, provided bioinformatics expertise, contributed to the methodology, and supervised the work. All three authors contributed to the paper, with a majority written by BT.

## Supplementary Material

Additional File 1 Literature-derived HLA-A*0201 binders and non-binders. List of HLA-A*0201 binding and non-binding peptides gathered from the literature. The papers from which these peptides were derived are cited in the text. Click here for file

Additional File 2 HLA-B*3501 LDA Q-Q plot. Q-Q plot showing distribution of LDA scores for the H-2Kd dataset from the community binding resource. The horizontal axis has been scaled according to normal probabilities, so that points from a normally distributed variable would fall along a straight line (shown in blue). Scores lying above the thresholds indicated would be classified as epitopes. The realized sensitivity of 0.44 for a specificity of 0.95 is indicated as the proportion of epitopes whose scores lie above the threshold of 0.95. Of the four datasets used, this one deviates most strongly from normality. Click here for file

Additional File 3 H-2Kd LDA Q-Q plot. Q-Q plot showing distribution of LDA scores for the H-2Kd dataset from the community binding resource. The horizontal axis has been scaled according to normal probabilities, so that points from a normally distributed variable would fall along a straight line (shown in blue). Scores lying above the thresholds indicated would be classified as epitopes. The realized sensitivity of 0.42 for a specificity of 0.99 is indicated as the proportion of epitopes whose scores lie above the threshold of 0.99. Only the nominal values for specificity are used, since the actual ones coincide or are better. Click here for file

Additional File 4 HLA-A*0201 (literature) LDA Q-Q plot. Q-Q plot showing distribution of LDA scores for the HLA-A*0201 dataset derived from literature. The horizontal axis has been scaled according to normal probabilities, so that points from a normally distributed variable would fall along a straight line (shown in blue). Scores lying above the thresholds indicated would be classified as epitopes. The realized sensitivity of 0.33 for a specificity of 0.95 is indicated as the proportion of epitopes whose scores lie above the threshold of 0.95. Of the four datasets used, this one best fits the normality assumption. Click here for file
