# Supplementary material for: Strength in numbers: achieving greater accuracy in MHC-I binding prediction by combining the results from multiple prediction tools
Source: Immunome Res. 2007 Mar 24;3:5. doi: 10.1186/1745-7580-3-5 (PMC1847428; doi:10.1186/1745-7580-3-5)
Supplement: Additional File 1 — Literature-derived HLA-A*0201 binders and non-binders. List of HLA-A*0201 binding and non-binding peptides gathered from the literature. The papers from which these peptides were derived are cited in the text. [file 1745-7580-3-5-S1.pdf]

Additional File 1: List of HLA-A*0201 binding and non-binding peptides gathered from the literature.

Binders			Nonbinders		
ALAIAALEL	ALGFCGHTV	ALSLAAVLV	AFLSVKKQF	AIVFDHYDV	ALKNNGKVV
ALVEFEDVL	ASLDSDPWL	ATNQGRVEI	AMFQDPQER	AVLVVMACL	CFFCFKELE
ATVGIMIGV	CLTSTVQLV	CQLAKTCPV	DIKMILKMV	DLAQCFFCF	DLFLKEGAC
CTPERMAEA	DLLSHAFFA	DVWSFGILL	DLVFDECGI	EAGFIHCPT	ELCDNPFFA
FAFRDLCIV	FCFKELEGW	FLARLHAAA	ELFPPLFMA	ELTLGEFLK	EPDLAQCFF
FLDPRPLTV	FLEGCACTP	FLKDHRIST	FANSKFTLV	FEELTLGEF	FFCFKELEG
FLKLDRERA	FLWGPRALV	FTLVAPVSI	FIFLLFLTL	FLGGGGAGI	FTCLPLGVL
FTSAVLLLV	GILGFVFTL	GLCTLVAML	FVYVNGKKI	GAPTLPPAW	GITDDQLLA
GLIQLVEGV	GLKAGVIAV	GLLFLHTRT	GLTFKGPGA	GLYDPDCDE	GVLMKGQHL
GLLLAAATA	GTLGIVCPI	HLYQGCQVV	GVQGGFVSA	HACWPAFTV	HRISTFKNW
IISLWDQSL	ILGELREKV	ILHNGAYSL	IAKETNNKK	IILNGSLLT	IITTDNTFV
ILKEPVHGV	ILLRDAGLV	ILPRKHHRI	ILLCCMTSC	IVQENNGAV	KALETDVPA
IMIGVLVGV	ISTFKNWPF	KASEYLQLV	KDHRISTFK	KHSSGCAFL	KIAKETNNK
KIFGSLAPL	KIWEELSVL	KLDRERAKN	KLPDDFMGC	KMILKMVQL	KVKRKKNVL
KLPQLCTEL	KLTPLCVTL	KLVERLGAA	LAAVLVVMA	LAQCFFCIK	LLFNKVTLA
KLYCSYEVA	KNWPFLEGC	KQFEELTLG	LLGLWGTAA	LLLQYGSFC	LLPRSDLAV
KVAELVHFL	KVRRAIEQL	KVVSLVILA	LLQYGSFCT	LLTDDMIAA	LLTTSGVSA
LIADFLSGL	LLDSAVQNL	LLFTRYPDV	LLVGYNDSA	LPPAWQPFL	LQTTIHDII
LLMGTLGIV	LLNATDIAV	LLQAEAPRL	LVELAGQSL	LVFDECGIT	LVYVNGVVV
LLQYWSQEL	LMNNAFEWI	LTLGEFLKL	MACLVPAAT	MMSCSSEAT	MTEQTDEAV
MAEAGFIHC	MGAPTLPPA	MLDLQPETT	MTYTGGVMT	NLPYVLAFL	NVLLYNRLL
MMDAFILSA	MTSCVSEQL	NLNESLIDL	PDLAQCFFC	PLNPGPEAL	PLQALLERA
NLTHVLYPV	QIIGYVIGT	QLLALLPSL	PPAWQPFLK	QCFFCFKEL	QLDHGVLLV
QLQGYCASL	QVLAFGLLL	RAIEQLAAM	QLGREKGPI	QTLKAMVQA	QVHSSLSVV
RIDITLSSV	RIIYDRKFL	RLAEYQAYI	RALSLAAVL	RIILNGSLL	RLGSCPIVV
RLFWVWLLV	RLLSPTTIV	RLNEVAKNL	RLHLWLSDM	RLQSLQTYV	RVFTSAVLL
RLQEWCSVI	RMAEAGFIH	RVIEVLQRA	SGCAFLSVK	SIEDLEVTC	SIHVTVSNV
SIISAVVGI	SISALQSLL	SLLQHLIGL	SILLRDAGL	SLKRTGHGV	SLSVVRPMT
SLVDVMPWL	SLYWSHEAL	SMNATLVQA	SLVYVNGVV	STEAEQPFI	STHCSGPSV
SSGCAFLSV	STCWCVNTA	STFKNWPFL	STSRVPYDI	TMNNKSQSV	TVTTITVYA
SVFRENLFL	TLAKFSPYL	TLGEFLKLD	VAVNGPVAV	VLNDILSRL	VLRLCCKKL
TLGIVCPIC	TLHEYMLDL	TLLYATVEV	VLYQDVNCT	VTCTWKLPT	VTLPTGQCL
TLPPAWQPF	VLDGLDVLL	VLYGPDTPI	VVAGIVVLV	VVFLHVTYV	VVVIAVVAG
VMACLVPAA	VMMSCSSEA	WIIKNSWTA	VVYENQKQI	WAFSAIGNV	WIVQENNGA
WLQYFPNPV	WLSDCGEAL	WLWYIKIFI	WMTYTGGVM	WPFLEGCAC	WTVWSGNRA
YLHARLREL	YLSGANLNL	YMLDLQPET	YACFVSNLA	YATVEVPSL	YVCGIQNSV
