# Supplementary figures and images for: Strength in numbers: achieving greater accuracy in MHC-I binding prediction by combining the results from multiple prediction tools
Source: Immunome Res. 2007 Mar 24;3:5. doi: 10.1186/1745-7580-3-5 (PMC1847428; doi:10.1186/1745-7580-3-5)

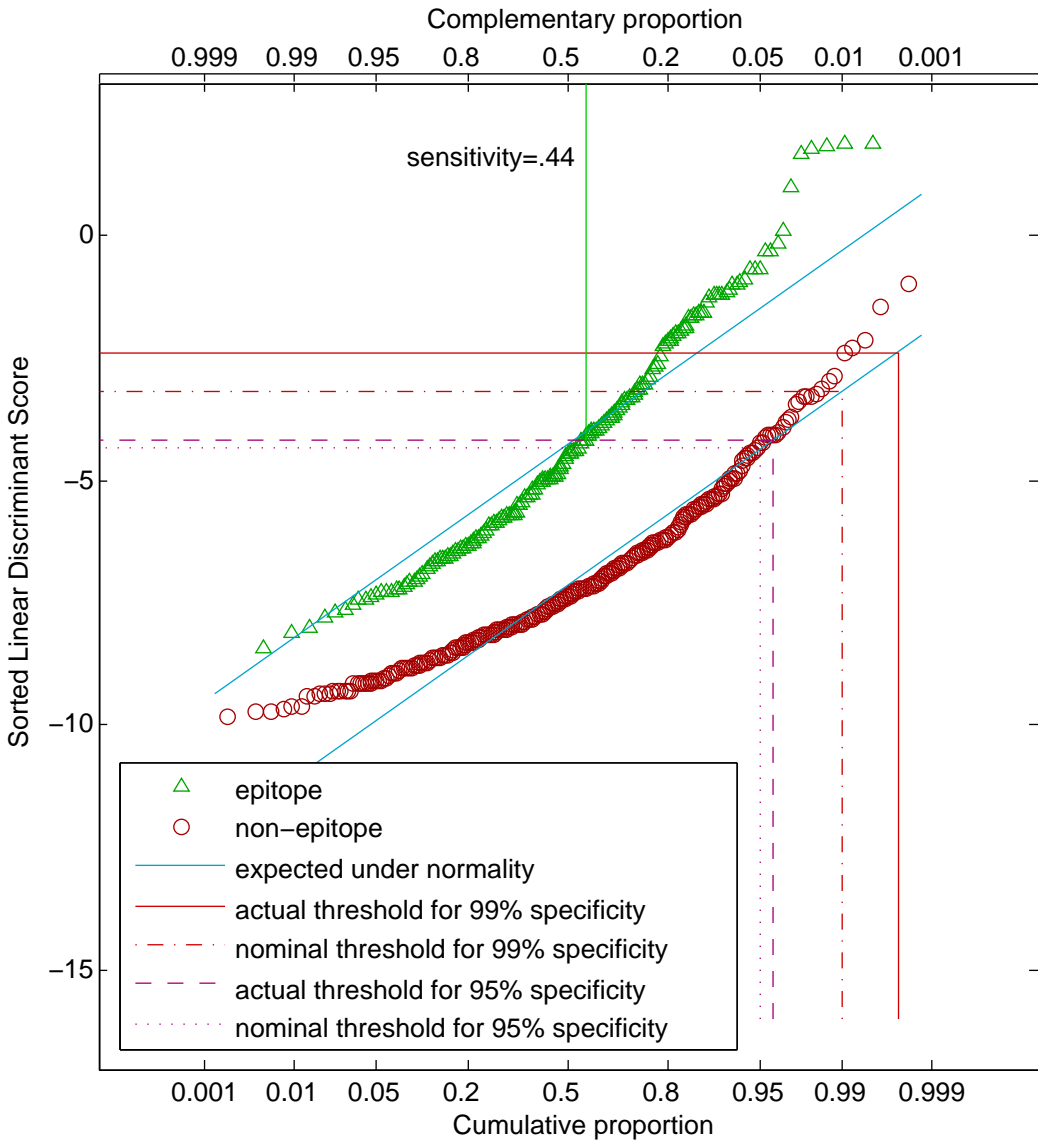

Supplement: Additional File 2 — HLA-B*3501 LDA Q-Q plot. Q-Q plot showing distribution of LDA scores for the H-2Kd dataset from the community binding resource. The horizontal axis has been scaled according to normal probabilities, so that points from a normally distributed variable would fall along a straight line (shown in blue). Scores lying above the thresholds indicated would be classified as epitopes. The realized sensitivity of 0.44 for a specificity of 0.95 is indicated as the proportion of epitopes whose scores lie above the threshold of 0.95. Of the four datasets used, this one deviates most strongly from normality. [file 1745-7580-3-5-S2.pdf]

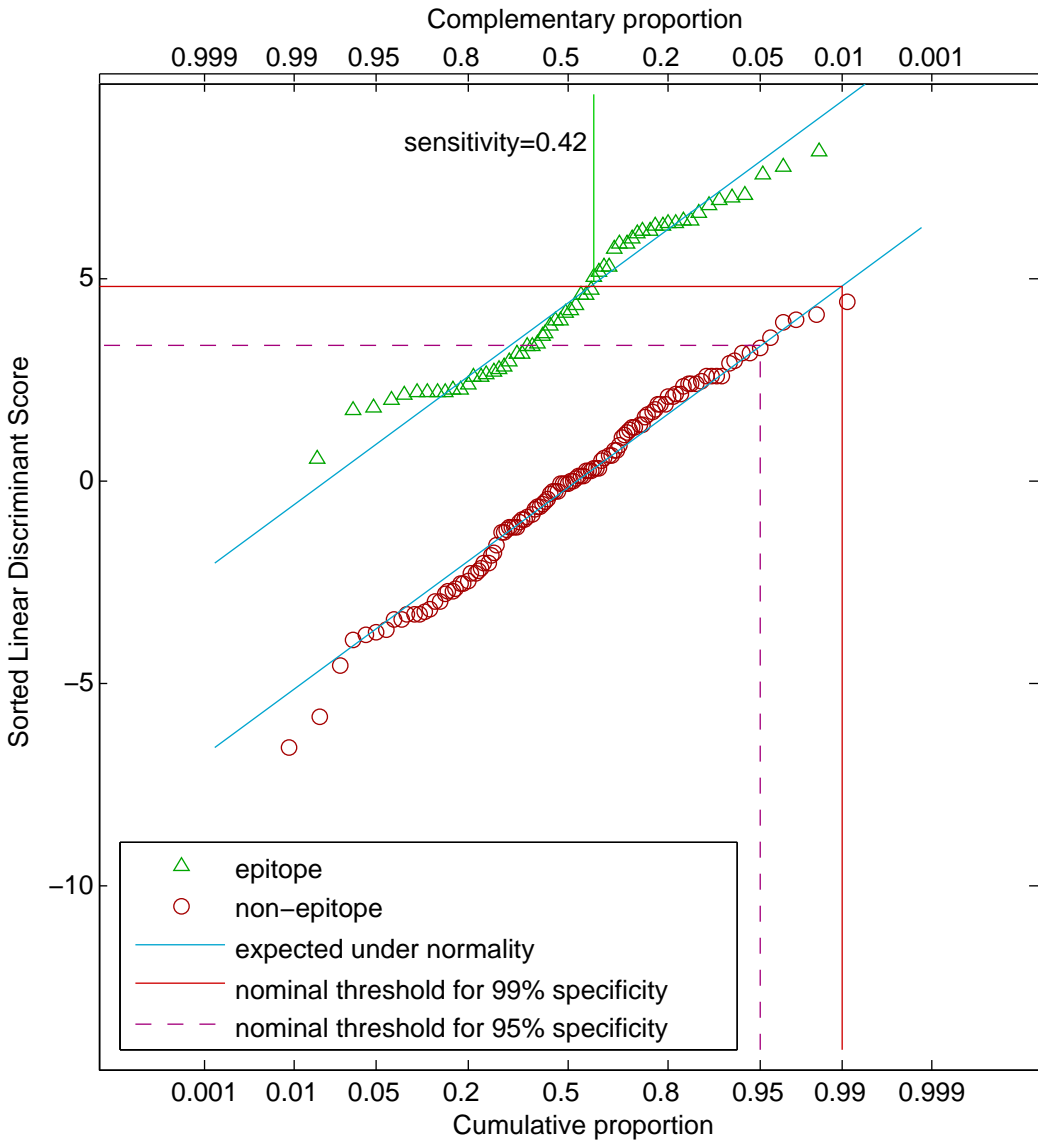

Supplement: Additional File 3 — H-2Kd LDA Q-Q plot. Q-Q plot showing distribution of LDA scores for the H-2Kd dataset from the community binding resource. The horizontal axis has been scaled according to normal probabilities, so that points from a normally distributed variable would fall along a straight line (shown in blue). Scores lying above the thresholds indicated would be classified as epitopes. The realized sensitivity of 0.42 for a specificity of 0.99 is indicated as the proportion of epitopes whose scores lie above the threshold of 0.99. Only the nominal values for specificity are used, since the actual ones coincide or are better. [file 1745-7580-3-5-S3.pdf]

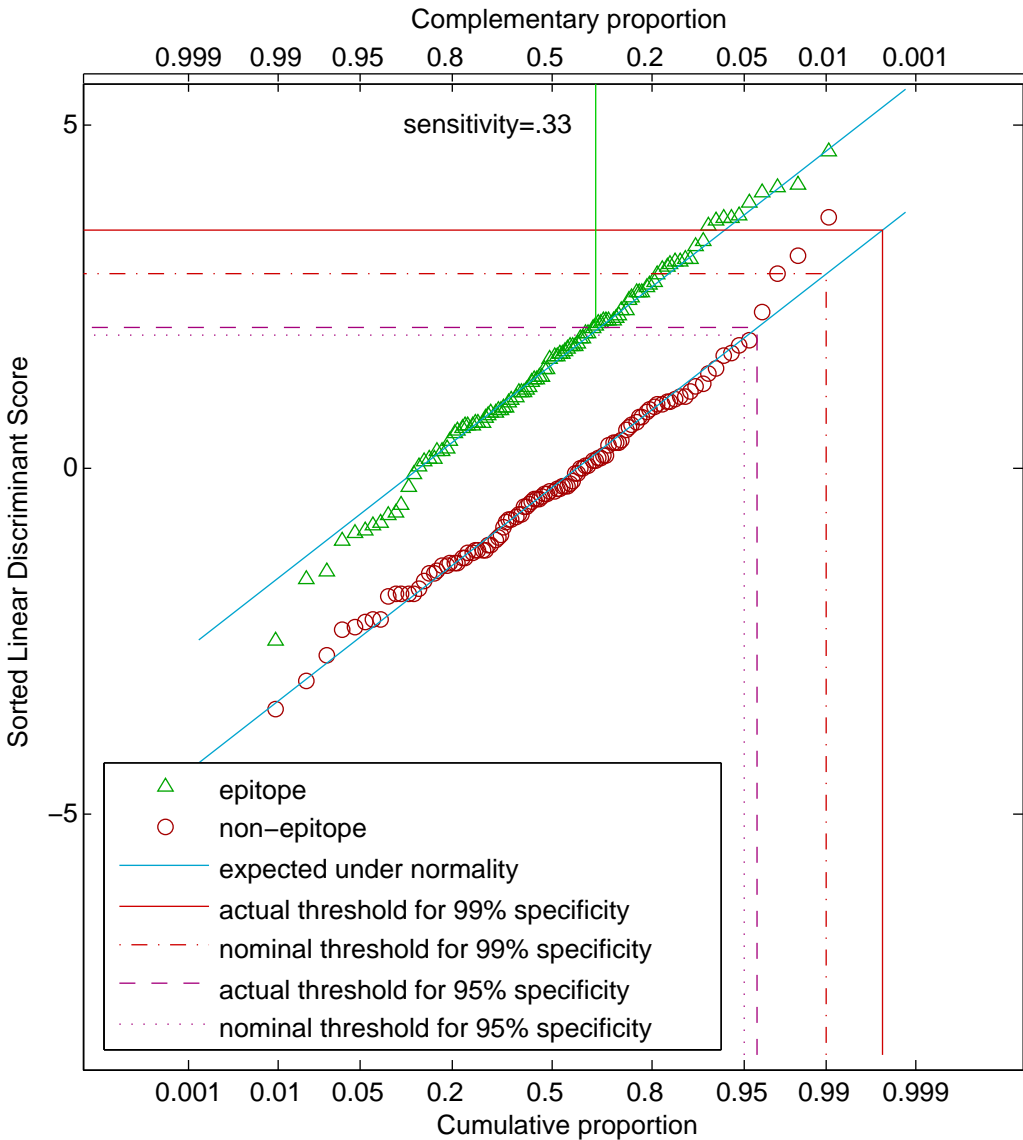

Supplement: Additional File 4 — HLA-A*0201 (literature) LDA Q-Q plot. Q-Q plot showing distribution of LDA scores for the HLA-A*0201 dataset derived from literature. The horizontal axis has been scaled according to normal probabilities, so that points from a normally distributed variable would fall along a straight line (shown in blue). Scores lying above the thresholds indicated would be classified as epitopes. The realized sensitivity of 0.33 for a specificity of 0.95 is indicated as the proportion of epitopes whose scores lie above the threshold of 0.95. Of the four datasets used, this one best fits the normality assumption. [file 1745-7580-3-5-S4.pdf]
